# Synthetic Gene Circuits Enable Sensing in Engineered Living Materials

**DOI:** 10.3390/bios15090556

**Published:** 2025-08-22

**Authors:** Yaxuan Cai, Yujie Wang, Shengbiao Hu

**Affiliations:** 1 Hunan Provincial Key Laboratory of Microbial Molecular Biology, College of Life Science, Hunan Normal University, No. 36 Lushan Street, Changsha 410081, China; caiyaxuan@hunnu.edu.cn; 2 Department of Pharmaceutical Engineering, China Pharmaceutical University, No. 639 Longmian Avenue, Nanjing 211198, China

**Keywords:** engineered living materials, gene circuits, signal transduction, biosensing

## Abstract

Engineered living materials (ELMs) integrate living cells—such as bacteria, yeast, or mammalian cells—with synthetic matrices to create responsive, adaptive systems for sensing and actuation. Among ELMs, those endowed with sensing capabilities are gaining increasing attention for applications in environmental monitoring, biomedicine, and smart infrastructure. Central to these sensing functions are synthetic gene circuits, which enable cells to detect and respond to specific signals. This mini-review focuses on recent advances in sensing ELMs empowered by synthetic gene circuits. Here, we highlight how rationally designed genetic circuits enable living materials to sense and respond to diverse inputs—including environmental chemicals, light, heat, and mechanical loadings—via programmable signal transduction and tailored output behaviors. Input signals are classified by their source and physicochemical properties, including synthetic inducers, environmental chemicals, light, thermal, mechanical, and electrical signals. Particular emphasis is placed on the integration of genetically engineered microbial cells with hydrogels and other functional scaffolds to construct robust and tunable sensing platforms. Finally, we discuss the current challenges and future opportunities in this rapidly evolving field, providing insights to guide the rational design of next-generation sensing ELMs.

## 1. Introduction

Genetic circuit-based whole-cell biosensors have emerged as powerful tools in synthetic biology, enabling living cells to detect specific analytes and convert these detections into measurable signals [1,2]. These systems typically involve the integration of sensing elements, signal processing modules, and output components such as fluorescent proteins or pigments into the host genome through genetic engineering [3]. Upon exposure to external stimuli, including synthetic inducers or environmental factors, these biosensors activate gene expression pathways, such as transcription factor or riboswitch-mediated mechanisms, to generate quantifiable biological responses [4]. Their modularity, programmability, and compatibility with cellular replication render them particularly attractive as versatile sensing platforms, capable of detecting diverse analytes such as heavy metals, antibiotics, or metabolites, and converting these detections into outputs like fluorescence, color change, or odor, as demonstrated in recent studies [5,6,7,8].

Despite these advantages, traditional whole-cell biosensors face challenges in stability, portability, and performance under real-world conditions—for example, fluctuating environmental factors such as temperature, pH, and humidity, biological interference from complex sample matrices or contaminants, and operational constraints in field deployment compared to controlled laboratory settings [9,10]. To address these limitations, researchers have developed a new class of materials known as ELMs, which combine living cells with synthetic matrices such as hydrogels, polymers, and inorganic scaffolds [11,12]. These cellularly integrated systems allow for the structural confinement and protection of cells, enhance the stability of biological outputs, and provide improved mechanical and environmental resilience [13,14,15]. By integrating biology with materials science, ELMs create programmable platforms for engineering dynamically responsive functional materials [16,17].

**Figure 1:**
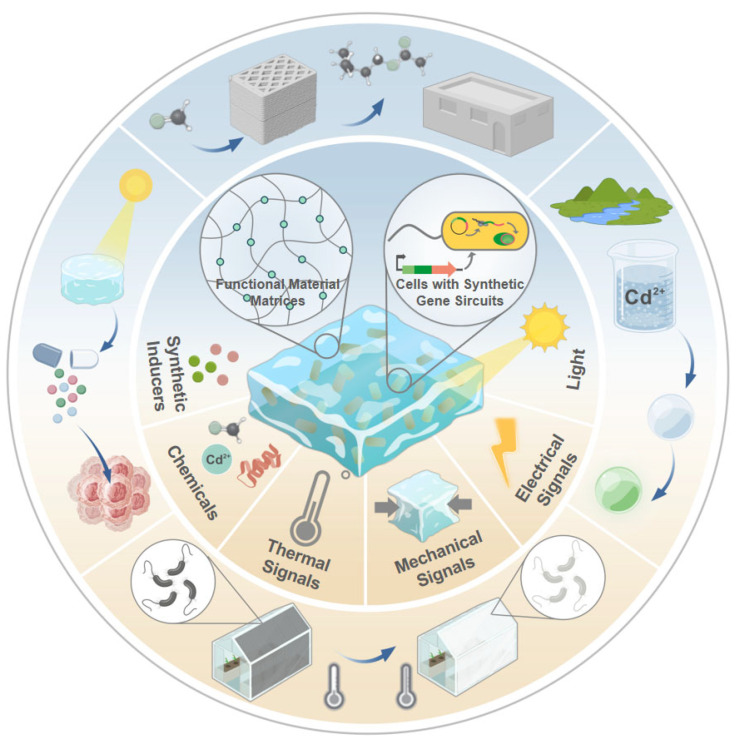
Engineered Living Materials (ELMs) utilize synthetic gene circuits to sense and convert stimuli into functional outputs. This framework encompasses various input signals (synthetic inducers, environmental chemicals, light, thermal, mechanical, and electrical signals) and enables applications in environmental monitoring, biomedicine, and smart infrastructure. Created with BioGDP.com. Reproduced with permission from [26], copyright © 2025 BioGDP.

Sensing ELMs have gained increasing attention for their ability to detect and respond to environmental signals. By embedding synthetic gene circuits within microbial or mammalian cells, these systems can be programmed to perform complex sensing tasks [16,18]. Compared to conventional materials, sensing ELMs offer superior adaptability and responsiveness to a broad range of stimuli, including chemical, optical, thermal, electrical, and mechanical stimuli (Figure 1). With emerging applications in environmental monitoring, precision medicine, industrial biosafety, and smart infrastructure [19,20,21]. While previous studies have explored the use of ELMs in stimulus-responsive systems [22], biological therapy [23,24], and sustainable biomanufacturing [25], they have largely focused on material fabrication and the integration of natural cells, with comparatively scant attention to the rational design of sensing functions using synthetic gene circuits.

In this mini review, we focus on recent progress in the development of sensing ELMs driven by genetic circuit design. We categorize input signals (e.g., light, ions, molecules) based on their source and physicochemical properties and highlight representative sensing systems—primarily proof-of-concept demonstrations illustrating key design principles, alongside selected examples tested under application-relevant conditions—that exemplify each type (Table 1). Special emphasis is placed on circuit construction principles, signal transduction mechanisms, and the coupling of biological function with material structure. Finally, we discuss current limitations such as functional stability, biosafety (e.g., preventing horizontal gene transfer), and control of expression accuracy, and propose future directions for building the next generation of programmable and responsive living materials.

## 2. Synthetic Inducer–Rsponsive ELMs

Synthetic inducers here refer to artificial small molecules deliberately added in laboratory settings to activate genetic circuits, in contrast to environmental chemicals that originate naturally (e.g., metal ions, gases, metabolites). During the early stages of synthetic biology development, researchers commonly utilized artificial classic small-molecule inducers like isopropyl β-D-1-thiogalactopyranoside (IPTG) and anhydrotetracycline (aTc)**,** which are necessary manual additions to establish standardized and controllable gene expression systems [47,48]. IPTG is a fundamental inducer commonly employed to modulate gene expression in *E. coli*. In the absence of IPTG, the lac repressor protein binds to the lac operon, hindering RNA polymerase from attaching to the promoter and thus repressing gene expression [49,50,51]. Consequently, by designing gene switches utilizing the lac promoter (e.g., Tet-on and Tet-off systems), precise control of target gene expression can be achieved [52].

**Figure 2:**
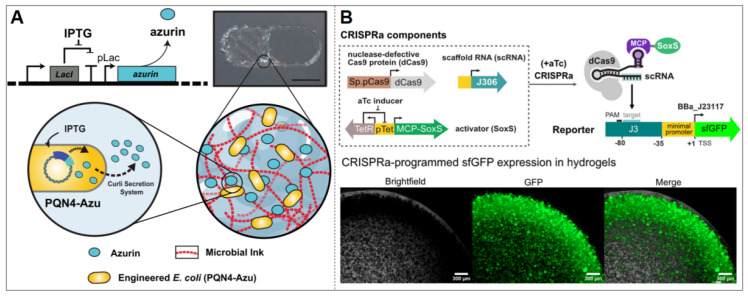
Synthetic Inducer–Responsive ELMs. (**A**) IPTG-inducible *E. coli* engineered to express and secrete the anticancer protein azurin. Reproduced with permission from [28], copyright © 2021, Anna M. Duraj-Thatte et al. (**B**) aTc-responsive *E. coli* encapsulated in F127-BUM hydrogel expressing sfGFP. Reproduced with permission from [17], copyright © 2023, Elsevier Ltd. (Amsterdam, The Netherlands).

In a separate investigation of a controllable drug-release ELM, Duraj-Thatte et al. designed an IPTG-inducible expression system for the anticancer protein azurin, a redox protein of bacterial origin (Figure 2A) [28]. This system employed pET21d as the vector, incorporating an IPTG-responsive element (LacI/PT7) and an azurin expression module. Additionally, it retained the N-terminal SEC signal peptide and the N22 targeting sequence specific to the curli system, facilitating efficient extracellular transport of azurin through the curli secretion channel. This synthetic circuit was introduced into the engineered *E. coli* strain PQN4 and coordinated with the microbial ink system expressing the structural proteins CsgA-α/γ [28]. The functional ELM was formed through 3D printing technology, achieving efficient and spatially controllable release of the therapeutic protein azurin. Beyond *E. coli*-based systems, González et al. demonstrated that IPTG-inducible genetic circuits can also be constructed in Bacillus subtilis spores, where the responsive module LacI/PT7 drives the expression of reporter proteins such as GFP. The resulting spore-based ELMs exhibited robust stability and could be 3D printed into resilient living materials capable of sensing and reporting environmental cues [30].

Another established synthetic small-molecule inducer is aTc. The induction mechanism of aTc primarily hinges on the interaction between the tetracycline-responsive protein (TetR) and the promoter region (tetO). In the absence of the inducer, TetR binds to tetO, impeding the initiation of RNA polymerase transcription. Upon the introduction of aTc, TetR associates with aTc, consequently alleviating the promoter’s suppression and facilitating gene expression [53]. Specifically within sensing ELMs, aTc prompts the expression of target genes by activating designated gene expression systems, such as the CRISPRa program [54,55].

Sugianto et al. developed an aTc-responsive *E. coli* ELM by employing the CRISPRa program to induce the expression of the target gene sfGFP [17] (Figure 2B). This system utilizes the pTet promoter to produce the MCP–SoxS activator protein. It enhances the transcriptional activity of the downstream promoter and drives the expression of sfGFP. Encapsulation of synthetic gene circuit-bearing *E. coli* within thermosensitive F127-BUM hydrogel demonstrated sustained system activation, maintaining functional stability through 19-day induction delays while preserving 97% of initial sfGFP expression levels across multiple induction cycles [17].

## 3. Chemical-Responsive ELMs

Environmental chemical inputs (e.g., metal ions, gases, metabolites) stand out as a prevalent form of signal input in current sensing ELMs. This approach typically employs specific chemical species as triggers, translating input signals into observable outputs like fluorescence, odor, and proteins [29,37,56]. Compared to systems requiring deliberate addition of synthetic inducers, this ELM system enables direct environmental analyte detection without requiring external intervention, while minimizing ecological disturbance by eliminating the need to introduce non-native chemical inducers into natural environments, thereby preventing potential ecological disruption. These characteristics make it particularly suitable for environmental monitoring and biomedical applications [57,58].

**Figure 3:**
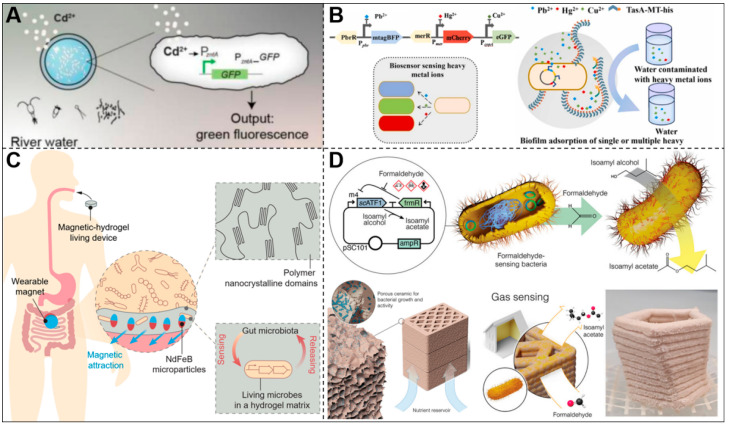
Chemical-Responsive ELMs. (**A**) Cadmium-inducible genetic circuits embedded in a dual-network hydrogel matrix enable *E. coli* to express GFP for heavy metal detection. Reproduced with permission from [34], copyright © 2021, Tzu-Chieh Tang et al., under exclusive licence to Springer Nature America, Inc. (**B**) Schematic diagram of ELMs based on *B. subtilis* for heavy metal sensing. Reproduced with permission from [32], copyright © 2023 Elsevier B.V. (**C**) Magnetically retained hydrogel capsule encapsulating a heme-sensing probiotic strain for in situ gastrointestinal bleeding detection, illustration for conceptual purposes; current demonstration in mice. Reproduced with permission from [36], copyright© 2021 Wiley-VCH GmbH. (**D**) Porous ceramic composite encapsulating *E. coli* programmed to convert formaldehyde into banana-scented isopentyl acetate via acetyltransferase expression. Reproduced with permission from [37], copyright © 2024 André R. Studart, Daniel Zindel, Aline Mailard et al., Advanced Materials published by Wiley-VCH GmbH.

Cadmium ions (Cd^2+^), common heavy metal pollutants, pose significant risks due to their high toxicity, potential to damage human health (e.g., kidney damage), and to ecosystems through persistent water and soil pollution [59]. To address this, Tang et al. developed a genetically modified ELM responsive to metal ions (Figure 3A) [34]. This system employs a synthetic genetic circuit driven by the promoter PzntA (responsive to Zn^2+^, Pb^2+^, Cd^2+^) and the regulator ZntR, with GFP as the reporter [34]. While the promoter is broadly metal-responsive, Cd^2+^-specific activation was experimentally validated, demonstrating practical relevance. Engineered *E. coli* were encapsulated in alginate-polyacrylamide hydrogel and exposed to cyclic 50 ppm Cd^2+^ for 7 days, during which cells retained >90% viability and 85% signal fidelity [34]. It is conducive to achieving continuous monitoring and offering a promising strategy for environmental pollutant surveillance. Advancing beyond single-ion specificity, recent ELMs have been engineered for simultaneous detection of multiple heavy metals through integrated genetic circuits. For example, Zhu et al. developed a *B. subtilis* biofilm@biochar (BBC) system [32] (Figure 3B) by integrating three metal-specific sensing modules into a single plasmid (pDG-BGR): Ppbr (Pb^2+^-responsive), PcopA (Cu^2+^-responsive), and Pmer (Hg^2+^-responsive), each driving a distinct fluorescent reporter (mtagBFP, eGFP, mCherry). This single-plasmid design enabled high-specificity, concurrent sensing of Pb^2+^ (0.1–75 μM), Cu^2+^ (0.1–75 μM), and Hg^2+^ (0.01–3.5 μM) without cross-talk from other ions (e.g., Ni^2+^, Cd^2+^, Zn^2+^) [32]. This integrated approach exemplifies a coordinated multi-ion response critical for complex pollution scenarios.

For in vivo disease monitoring, Liu et al. constructed a magnetic hydrogel–based ELM capable of detecting heme—a biomarker of gastrointestinal bleeding—in the gastrointestinal tract in a murine model [36] (Figure 3C). The design uses a circuit with the heme-sensitive repressor HrtR as the sensor and the PL (HrtO)-luxCDABE as the reporter, producing a bioluminescent signal upon heme detection [60]. Unlike conventional fecal occult blood tests that detect bleeding indirectly after excretion, this system enables real-time, site-specific monitoring in the intestinal lumen. Specificity is ensured because physiological heme levels in the gut are very low (<1 μM), whereas pathological bleeding releases >10 μM heme [36]. These engineered bacteria were enclosed within a pliable and biocompatible magnetic hydrogel matrix, which incorporates magnetic nanoparticles to enable positioning by an external magnet. This magnetic control allowed the hydrogel to be directionally retained in the intestine for up to 7 days, far exceeding the typical 6–48 h gastrointestinal transit time of conventional ingestible devices [36]. This minimally invasive strategy (requiring ingestion but no surgery) is a convenient approach for localized monitoring of intestinal bleeding.

Beyond ions and biological molecules, gas molecules can also be exploited as human-readable outputs in sensing ELMs. Researchers have developed an ELM to detect formaldehyde, a common toxic volatile compound in building materials and household products (Figure 3D) [37]. This system employed a modular genetic circuit based on the formaldehyde detoxification pathway of *E. coli*, using the formaldehyde-specific transcriptional repressor FrmR together with its regulated promoter m4 (from plasmid pTR47m4). Upon formaldehyde exposure, inhibition by FrmR is relieved, and the m4 promoter drives the expression of acetyltransferase ATF1. ATF1 then catalyzes the esterification of isopentanol with acetyl-CoA, producing isoamyl acetate (IAct), a distinct, harmless banana-like odor, serving as an easily perceivable and benign signal for most individuals [37]. The engineered *E. coli* were embedded in a porous ceramic material with efficient nutrient distribution, high mechanical strength, and structural stability. Experiments demonstrate that this system can detect formaldehyde at concentrations as low as 0.12 ppm—approximately 8 times more sensitive than the average human olfactory threshold—and achieve the volatilization and release of IAct within 3 h, indicating high sensitivity and efficiency [37]. Notably, individuals with olfactory impairments cannot directly utilize this signal. It can be improved by replacing ATF1 with fluorescent reporters (e.g., sfGFP) in the genetic circuit to enable visual formaldehyde detection.

## 4. Light-Responsive ELMs

Light stimuli represent a highly controllable input modality that has attracted growing attention in the context of ELMs. To create genetic circuits that react to specific light wavelengths, optogenetic approaches commonly employ photosensitive proteins as fundamental sensing components, such as photoreductases or light-regulated transcription factors [61,62]. This technique provides several advantages, including rapid response, adjustability, and high spatiotemporal resolution, making it well-suited for regulating the adaptive behavior of living materials [63]. As optogenetic tools continue to advance, their application has expanded beyond the biomedical realm [64,65] into biosensing and smart materials development [66], emerging as a crucial branch in contemporary ELMs research.

**Figure 4:**
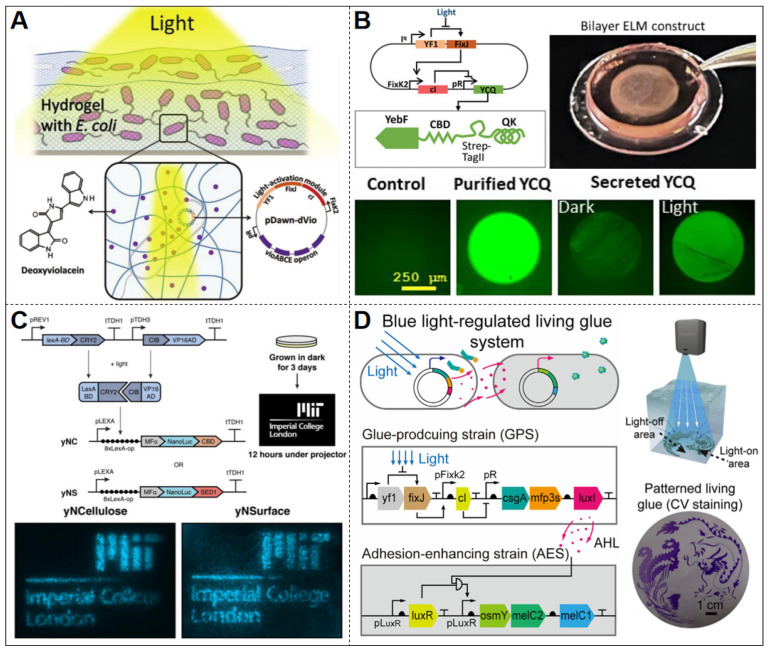
Light-Responsive ELMs. (**A**) Optogenetically Controlled Release of Anticancer deoxyviolacein from Engineered Hydrogels. Reproduced with permission from [42], copyright © 2018 WILEY-VCH Verlag GmbH & Co. KGaA, Weinheim. (**B**) Hydrogel-based light-activated delivery system with engineered *E. coli* secreting VEGF-mimetic peptide QK for angiogenesis induction. Reproduced with permission from [39], copyright © 2023 The Shrikrishnan Sankaran, Varun Sai Tadimarri, Priyanka Dhakane. Advanced Functional Materials published by Wiley-VCH GmbH. (**C**) Syn-SCOBY hybrid membrane integrating engineered yeast for NanoLuc expression under blue light, enabling optogenetic spatial patterning. Reproduced with permission from [38], copyright © 2021, The Charlie Gilbert et al., under exclusive licence to Springer Nature Limited. (**D**) Photoactivated Microbial Adhesive Circuit Enables Site-Specific Crack Repair via Protein-Directed Microsphere Aggregation. Reproduced with permission from [41], copyright © 2020 Elsevier Inc.

Light-responsive ELMs have emerged as a promising approach for light-controlled drug release. Sankaran et al. developed a light-sensitive ELM system capable of controllable synthesis and secretion of the antibacterial and antitumor drug deoxyviolacein (dVio) activated by blue light components in ambient light [42] (Figure 4A). The system was engineered by introducing a blue light-sensing genetic circuit (pDawn-dVio) into endotoxin-free *E. coli* (ClearColi), which was then integrated with a hydrogel matrix. Similarly, Dhakane et al. employed the light-inducible vector pDawn to create a light-responsive material that releases pro-angiogenic factors [39] (Figure 4B). This system includes a transduction module that controls gene expression via light-sensitive transcription factors and an output module that secretes the active fusion protein YCQ (YebF-collagen-binding domain-QK fusion protein). YCQ incorporates the vascular endothelial growth factor (VEGF) mimetic peptide QK, which can stimulate angiogenesis by activating signaling pathways in vascular endothelial cells [67]. This function is critically relevant for treating peripheral vascular disease (PVD), where promoting new blood vessel formation restores oxygen supply to hypoxic tissues. To enhance system safety and material stability, Dhakane et al. implemented a double-layer hydrogel structure composed of 30% w/v Pluronic F127-diacrylate (PluDA) hydrogels. The inner layer encapsulates engineered bacteria in a hydrated, biocompatible environment to maintain their viability and function. The outer shell layer, also made of PluDA, acts purely as a protective barrier. Experimental results show that this system can consistently express and secrete the YCQ protein when induced by blue light, exhibiting strong light control. Additionally, it enables precise dosage adjustment (1 to 20 nM) based on light intensity and sustains stable release for up to 9 days, exceeding the functional duration of the magnetically retained ELM for heme detection, which demonstrated operational stability in vivo for 7 days (Section 3, Paragraph 3) [39].

Light-mediated patterning, as a spatiotemporally precise control strategy, endows ELMs with powerful capabilities for dynamically regulating cellular behaviors, generating functional architectures, and enabling intelligent responses [68,69,70]. Early pioneering work by Jin and Riedel-Kruse demonstrated that light could be used to guide bacterial deposition into defined spatial patterns by optogenetically controlling adhesin expression, thereby laying the foundation for subsequent optogenetic strategies in ELM construction [71]. Gilbert et al. devised an ELM construction approach utilizing a bacteria-yeast co-culture system known as Syn-SCOBY [38] (Figure 4C). This system involves incorporating engineered Saccharomyces cerevisiae into in situ-grown bacterial cellulose (BC). The engineered yeast—a yNSurface strain that displays NanoLuc on the yeast cell surface and yNCellulose, which secretes a NanoLuc-CBD—secretes functional enzymes within the BC network, facilitating autocatalytic properties of the material. The researchers implemented a light-responsive genetic circuit activated by blue light signals based on the CRY2-CIB dimer system. Engineered yeast expresses LexA-CRY2 and VP16-CIB proteins that form a heterodimer upon blue light exposure, initiating NanoLuc luciferase expression by binding to the LexA operator [38]. Optical patterning was achieved by projecting blue light through masks or using a projector, thereby creating spatially defined activation of NanoLuc expression within the BC membrane. This method enables the generation of user-defined patterns, offering an approach for spatially programmable functionalization of BC-based ELMs [38].

In a related development, An et al. engineered a light-responsive bio-glue based on the pDawn blue light induction system [41] (Figure 4D). By co-culturing glue-producing and signal-receiving cells and incorporating the adhesion protein CsgA-Mfp3s and the AHL (acyl-homoserine lactone) cell communication mechanism, they established a two-strain regulatory system. Researchers employed a digital light projector to emit precise blue light patterns, triggering gene expression exclusively in targeted areas. This localized activation induces the formation of a bioadhesive coating, allowing the ELM to demonstrate controllable adhesive properties in light-directed patterns. This material serves as a light-regulating adhesive, enabling predictable dynamic underwater adhesion and spatially resolved substrate patterns [41]. Such light-responsive platforms endow ELMs with programmable sensing and response capabilities, highlighting their potential in biosensing (e.g., light-regulated therapeutic secretion [39]), patterned material fabrication (e.g., spatially resolved bioadhesives [41] and optogenetic cellulose patterning [38]), and anti-counterfeiting applications (e.g., optically encoded drug-release systems [42]).

## 5. Other Stimuli–Responsive ELMs

Compared with chemical or light inputs, the development of sensing ELMs responsive to non-electromagnetic physical signals—such as thermal, mechanical, or electrical stimuli—remains relatively limited, with relatively few reported systems. Although thermosensitive elements like the TlpA36 promoter [72] can directly interface with gene circuits, universal transduction modules for mechanical and electrical signals—capable of reliable genetic circuit coupling—are still lacking. Current strategies primarily rely on signal transduction via materials or endogenous pathways to convert physical signals into intracellular biological signals, thereby enabling stimulus-responsive gene activation [73,74].

### 5.1. Temperature-Responsive ELMs

Temperature is a crucial physical factor that can profoundly impact protein conformational stability and enzyme activity. Temperature-responsive ELMs modulate gene expression to detect and respond to environmental temperature changes.

**Figure 5:**
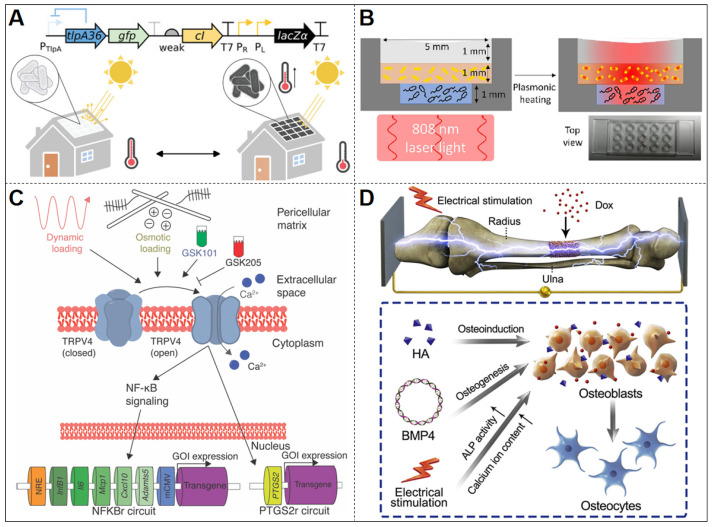
Other Physical Stimuli–Responsive ELMs. (**A**) Temperature-regulated genetic circuit in *E. coli* driving β-galactosidase-mediated pigment synthesis to modulate light absorption. Reproduced with permission from [44], copyright © 2023 Mikhail G. Shapiro, Julia A. Komfield, Michael A. Garrett et al., Advanced Science published by Wiley-VCH GmbH. (**B**) Schematic depiction of the bilayer hydrogel construct in a polymer well plate, which contained bacteria genetically modified to produce mCherry at temperatures above 39 °C. Reproduced with permission from [43], copyright © 2023 Elsevier B.V. (**C**) TRPV4-mediated mechanogenetic circuits in cartilage constructs detect osmotic stress and drive IL-1Ra production for inflammation-responsive therapy. Reproduced with permission from [45], copyright © 2021 Robert J. Nims, Lara Pferdehirt, Noelani B. Ho, Alireza Savadipour et al., some rights reserved; exclusive licensee American Association for the Advancement of Science. (**D**) Programmable Bone Regeneration Platform Combining Electroresponsive BMP-4 Plasmid Delivery with Doxycycline-Inducible Transgene Control. Reproduced with permission from [46], copyright © 2019 Elsevier Ltd.

Xiong et al. developed a thermoregulated genetic circuit in ELMs to control production of a black light-absorptive chromophore [44] (Figure 5A). The system utilizes the temperature-sensitive repressor TlpA36 as a molecular thermostat, creating a transcriptional feedback loop: (1) Below the activation threshold of 36 °C, TlpA36-mediated repression of cI enables PR/PL-driven LacZ expression; (2) At elevated temperatures above 36 °C, TlpA36 inactivation permits cI accumulation, suppressing downstream LacZ production through promoter inhibition. To produce light-absorbing pigments, the system utilizes the S-gal substrate, which is converted into esculetin by β-galactosidase. Esculetin then complexes with Fe^3+^ to generate a highly absorbent black product. Additionally, the fluorescent protein mWasabi is incorporated into the system to indicate gene expression in high-temperature regions, enhancing visualization. Engineered bacteria are immobilized on a polycarbonate membrane to create centimeter-scale ELM patches. At 32 °C, which is relatively low for optimal microbial growth, the system induces pigment production, elevating local membrane temperature. Conversely, at higher temperature (42 °C), the circuit suppresses pigment formation to prevent overheating [44]. This precise temperature modulation mechanism significantly enhances cell proliferation and material deposition, enabling autonomous regulation of growth homeostasis [44].

Building on a complementary strategy, Basaran et al. demonstrated a hybrid approach by embedding gold nanorods (AuNRs) within a bilayer hydrogel ELM to achieve NIR (near-infrared)-induced photothermal activation of engineered bacteria (Figure 5B) [43]. Upon 808 nm laser irradiation (0.5–0.7 W/cm^2^), the AuNRs generated a localized temperature increase of 10–29 °C (steady-state, depending on laser power and AuNR concentration), which activated a TlpA39 thermosensitive promoter (switching ON above 39 °C) to drive mCherry expression in ClearColi BL21 [43]. In contrast to the autonomous feedback regulation seen in the TlpA36 circuit, this design integrates an external physical component—AuNR-mediated photothermal conversion—to achieve remote and spatially resolved control of gene expression. The system exhibited a sharp thermal threshold, providing a tunable and externally addressable mode of thermal activation.

### 5.2. Mechanical Signal-Responsive ELMs

The majority of current mechanical-sensing ELMs utilize natural dinoflagellates [75,76], which are single-celled marine plankton known for their natural bioluminescence, and inherently detect mechanical stimuli and transduce them into visible light via luciferin oxidation. However, these natural systems depend on innate regulatory pathways, not on deliberately engineered synthetic genetic circuits. Therefore, as our focus is on ELMs specifically designed with programmable and targeted synthetic circuits, systems relying on natural dinoflagellates fall outside the scope of this discussion. Instead, we focus on genetically engineered mechanical signal-responsive ELMs, which sense mechanical loading through mechanosensitive ion channels and activate gene expression in response.

In ELMs, researchers commonly employ osmotic pressure-induced mechanical loading to activate mechanosensitive ion channels on cell membranes. This activation triggers a signaling cascade involving calcium ion influx, leading to the activation of downstream transcription factors and the initiation of synthetic gene circuits. Nims et al. designed an engineered cartilage construct where primary porcine chondrocytes—modified to express a TRPV4-mediated mechanosensitive gene circuit—sense mechanical loading or osmotic stress to trigger production of the anti-inflammatory protein IL-1Ra, effectively mitigating cartilage degradation in osteoarthritis models [45] (Figure 5C). Encapsulated within agarose hydrogels, this system activates selectively under cyclic stress through TRPV4 signaling pathways, demonstrating autonomous self-regulation of therapeutic production without external intervention. The circuit can sustain IL-1Ra expression over extended periods (e.g., T50% decay time of ~22 h in PTGS2r-based designs) and maintain function under inflammatory conditions to preserve tissue integrity [45]. This system provides both self-regulating therapeutic control and long-term functional stability, offering clear advantages over the conventional drug delivery approach.

### 5.3. Electrical Signal-Responsive ELMs

Electrical signals serve as a programmable input method for ELMs. While numerous ELMs leveraging electron transfer have been applied in fields such as bio-batteries, they fundamentally depend on cellular metabolites (e.g., lactic acid, CO_2_) as signaling molecules for electrochemical transduction [77,78,79,80]. Since this signal conversion relies on intermediate biochemical steps rather than direct electrical-genetic interfacing, such systems fall outside this section’s focus on electrical signals as direct input for genetic circuits. Currently, there is still a lack of systematic research on directly activating or inhibiting promoters via electric current or voltage to control genetic circuits. One innovative approach embeds engineered cells in electroresponsive materials, where electrical stimulation alters surface charge to release plasmids or molecules that then enter cells and activate downstream expression, enabling an electrical signal-responsive function [46].

Cui et al. developed an electrically dominated scaffold system for bone tissue regeneration, where electrical stimulation (ES) serves as the primary trigger for controlled plasmid release, while small molecules Dox act as auxiliary regulators of gene expression (Figure 5D) [46]. This scaffold immobilizes a polymer-DNA complex containing the plasmid encoding the osteoinductive factor BMP-4 (phBMP-4) on a conductive coating (PLA-AP). Initially, the polymer-DNA complex is securely attached to the conductive coating through electrostatic interactions. Upon an electrical pulse (ES), charge redistribution in PLA-AP induces interfacial polarization, weakening electrostatic bonds and facilitating plasmid release. The released plasmid is internalized by neighboring cells, leading to the expression of BMP-4 protein intracellularly and initiating osteogenic signaling. For auxiliary modulation, the researchers incorporated a Dox-responsive element to fine-tune BMP-4 expression when needed [46]. This modular regulatory circuit—with electrical pulses and Dox as the combined inputs controlling BMP-4 output—enables precise and dynamic control over the process of bone tissue regeneration.

## 6. Challenges and Potential Solutions

### 6.1. Functional Stability

Among the critical challenges facing sensing ELMs, functional stability—particularly long-term cell viability and signal consistency—demands primary attention as the cornerstone for reliable real-world deployment. ELMs rely on engineered cells for their core functionality, with their sensing capabilities dependent on protein and enzyme activities. Environmental changes, such as fluctuations in temperature and pH, can profoundly impact ELMs by compromising cell viability and destabilizing genetic circuit expression, which impairs sensing performance [27,75]. Harimoto et al. enhanced the stability of microbial surface proteins through the dynamic regulation of a synthetic gene circuit CAP (capsular polysaccharide) system, thereby improving their stability in the host [81]. Specifically, the programmable CAP coating protected engineered *E. coli* from extreme acidic conditions (pH 2.5) in the gastric environment, enabling them to resist gastric acid damage, and promote intestinal retention, thus prolonging the inflammation-sensing time.

Future advancements in enhancing engineered bacteria performance may be achieved through strategies like biofilm modifications—for example, modifying extracellular polymeric substance (EPS) composition to improve barrier properties against acidic or enzymatic degradation [11], or tuning viscoelastic stiffness to enhance resistance against acid, thermal, or mechanical stress [82,83]—to improve resistance to acid, temperature shifts, or mechanical stress—aimed at bolstering the environmental adaptability of ELMs.

### 6.2. Biosafety Enhancement

Beyond functional stability, biosafety concerns demand rigorous attention, with horizontal gene transfer (HGT) posing a primary risk. Engineered bacteria within ELMs may transfer resistance or pathogenic genes to native microorganisms, thereby disrupting ecological equilibrium [84]. Current strategies to mitigate HGT include gene silencing and synthetic auxotrophy design [85]. Gene silencing suppresses gene expression at the transcriptional or post-transcriptional level, preventing foreign or mobile genetic elements from being expressed in recipient cells [86,87]. Synthetic auxotrophy engineers microorganisms to depend on non-natural nutrients for survival, ensuring they cannot persist or function in natural environments [88,89,90]. Examples of these approaches encompass CRISPR-Cas-mediated targeted silencing [91,92,93], RNA interference technology [94,95], and the implementation of toxin/antitoxin systems [96].

In addition to genetic safeguards, physical biocontainment can be achieved through material design. When engineered cells are encapsulated within hydrogel-based ELMs, their potential leakage—particularly in flexible or stretchable devices—compromises sensing accuracy while raising biosafety and environmental concerns [13]. To tackle this challenge, Bhusari et al. employed Pluronic/Pluronic diacrylate (Plu/PluDA) hydrogels with tunable ratios of reversible and permanent covalent crosslinks to regulate bacterial growth and mobility [97]. Increasing the proportion of permanent crosslinks markedly reduced colony expansion, from 472 ± 439 µm^3^ to 213 ± 94 µm^3^ in mean volume after 6 h. Larger colonies (95th percentile) decreased from 1115 µm^3^ to 359 µm^3^ under the same conditions. These mechanical constraints slowed colony elongation, produced more compact and rounded morphologies, and homogenized colony size distribution, thereby minimizing the risk of bacterial migration and leakage. Future research can explore cell embedding and encapsulation techniques to mitigate cell leakage concerns.

### 6.3. Control of Expression Accuracy

In the absence of specific induction signals, engineered cells’ genetic circuits may still exhibit a basal level of target protein expression due to incomplete promoter repression or plasmid/genomic instability. Rather than a flaw, this basal activity reflects an inherent constraint of inducible systems and can become particularly relevant in light-responsive ELMs, such as the low-level expression of the YCQ protein in biomaterials engineered for light-regulated angiogenesis in the dark [39], and deoxyrubixanthin expression has been observed in ELMs for light-controlled drug release in the dark [42]. Although generally modest, such background expression can consume cellular resources and introduce noise into sensing signals, highlighting the importance of strategies to improve expression fidelity.

To address the issue of leaky protein expression, common strategies include implementing dual transcription-translation control mechanisms [98], which regulate target protein expression at both the transcription and translation levels. Additionally, in *E. coli*, a coherent feed-forward loop using a canonical amino acid suppressor tRNA has been developed to further reduce background expression [99]. This suppressor tRNA enables conditional readthrough of silent stop codons inserted into the target gene, so that translation only proceeds when the tRNA is expressed under inducer control, thereby minimizing leak while preserving high fold-induction.

## 7. Future Perspectives

### 7.1. Programmable Anti-Counterfeiting Designs

Current anti-counterfeiting research employs strategies like photoresponsive polymers [100] and 3D printing of specialized materials [101]. Significantly, these advanced techniques—optical patterning and 3D fabrication—directly align with the manufacturing strategies in ELMs [27,28,38]. By integrating the biorecognition capabilities of programmable genetic circuits with material structure encoding methods, ELMs can create environment-triggered, updatable, and hard-to-forge anti-counterfeiting solutions. In practice, two complementary fabrication strategies can be envisioned: (i) optical induction patterning, enabling rapid and reconfigurable generation of invisible QR codes or spatial patterns for on-demand authentication [102]; (ii) 3D bioprinting, allowing the construction of complex three-dimensional architectures with distinctive optical, spectral, or deformation characteristics for long-term, tamper-resistant tagging [101,103,104]. Combining these strategies enhances anti-counterfeiting complexity via embedded biosensing intelligence.

### 7.2. Genetic Encoding and Information Storage

Advances in biological information processing have enabled sensing ELMs to not only respond to stimuli but also store and retrieve encoded environmental information at the molecular level. A promising strategy involves the integration of DNA-based memory systems from synthetic biology to encode external signals into the genome or stable genetic modules [105,106]. Researchers have created intracellular “writers” like SCRIBE (Synthetic Cellular Recorders Integrating Biological Events) and DOMINO (DNA-based Ordered Memory and Iteration Network Operator). These tools use controllable recombinases or CRISPR-based editing systems to induce site-specific base changes or insertions in cellular DNA under certain stimuli, thereby encoding “historical records” genetically [107,108]. The same molecular platforms (e.g., CRISPR-Cas nucleases, base editors, recombinases) can also be integrated into gene circuits of ELMs, since their living-cell chassis provides a compatible environment for enzyme expression and regulation. And the types of stimuli used in such memory systems (e.g., light, small-molecule inducers) often overlap with those employed in sensing ELMs, making integration technically feasible [107]. By integrating such genetic storage tools with ELMs, these materials can generate response signals when exposed to environmental stimuli and convert external signals into biological codes, achieving the functions of information writing, reading, and even encrypted storage [106].

## 8. Discussion

While sensing ELMs continue to advance, important unresolved questions point to where the field could make its next major contributions. A key issue is how to further improve biosafety, long-term stability, and response accuracy in complex environments. As outlined earlier, strategies such as genetic safeguards and hydrogel encapsulation—already discussed in earlier sections—offer promising entry points, but achieving robust and predictable performance outside the laboratory still requires deeper innovation. Similarly, as noted above, direct electrical and mechanical responsiveness is still at a nascent stage, with most systems relying on indirect biochemical intermediates. Developing circuits that can be actuated directly by voltage, current, or mechanical force would unlock distinctive advantages that cannot be matched by conventional electrodes or mechanically sensitive devices. Another important direction concerns multi-signal integration. Beyond the validated examples of multi-metal detection, it remains an open challenge whether ELMs can be designed to couple fundamentally different classes of inputs—for instance, linking cytokine sensing with mechanical stress in tissue, or combining pH and toxin monitoring in environmental settings. Progress on these fronts will not only resolve key technical barriers but also expand the programmability and application scope of sensing ELMs.

## 9. Conclusions

In summary, the integration of synthetic biology and materials science has positioned sensing ELMs as a rapidly emerging and promising direction in next-generation biosensing technologies. By incorporating programmable genetic circuits into living cells and integrating them with material systems, these constructs enable autonomous sensing, precise control of gene expression, and specific functional responses. This review offers a structured overview of sensing ELMs, organized according to the nature of input signals, and highlights their diverse applications in areas such as environmental monitoring, medical diagnostics, and industrial sensing. Although challenges remain in ensuring biosafety, achieving high expression accuracy, and adapting to complex environments, continued advancements in genetic circuit optimization and material encapsulation strategies are steadily improving their reliability and versatility. As research progresses, sensing ELMs are expected to play a transformative role in fields including intelligent healthcare, environmental surveillance, secure information encoding, and unlocking unprecedented opportunities for bio-integrated technologies.

## Figures and Tables

**Table 1:** Genetic Circuit Design Specifications and Performance Specifications for Sensing ELMs.

Stimulus Type	Input Signal	Output Signal	Promoter	Reporter Gene	Host Organism	Material	Threshold	Stability	Ref.
**Synthetic Inducers**	IPTG*	RFP* (fluorescence)	PLac	RFP*	*E. coli*	hydrogel	0.1–1 mM	>72 h	[27]
	aTc*	RFP* (fluorescence)	PTet	RFP*	*E. coli*		50–200 ng/mL		
	VAI*	γ-PGA* (biopolymer)	Pspank(V)	γ-PGA*	*B. subtilis*		1 mM		
	VAI*	Catechol 2,3-dioxygenase (enzyme)	Pspank(V)	*xylE*	*B. subtilis*		1 mM		
	IPTG*	azurin (protein)	PLac	*Azurin*,	*E. coli*	CsgA*-αγ hydrogel	≥0.1 mM	effective one-time release upon induction	[28]
	IPTG*	endoribonuclease	PLac	*MazF*			≥0.1 mM		
	Ara*	RFP* (fluorescence)	ParaBAD	*mRFP1*	*E. coli*, *F. Gluconacetobacter hansenii*	BC*, gelatin	0.1% (*w*/*v*)	~5 days expt.	[29]
	aTc*	sfGFP* (fluorescence)	PTet	sfGFP*	*E. coli*	Pluronic F127-BUM hydrogel	0–100 ng/mL	>48 h	[17]
	IPTG*	GFP* (fluorescence)	Pspank(hy)	GFP*	*B. subtilis*	hydrogel	1 mM	>6 months	[30]
	xylose	GFP* (fluorescence)	Pxyl(s)	GFP*			1% *w*/*v*		
	vanillic acid	GFP* (fluorescence)	Pspank(V)	GFP*			1 mM		
	Cuminic acid	GFP* (fluorescence)	Pspank(C)	GFP*			0.5 mM		
	Theophylline	YFP* (fluorescence)	PconII	YFP*	*S. elongatus*	hydrogel	~0.5 mM	>7 days	[31]
**Chemicals**	Pb^2+^	mtagBFP* (fluorescence)	Ppbr	mtagBFP*	*B. subtilis*	biofilm@biochar	0.1 μg/L	>7 days	[32]
	Cu^2+^	eGFP (fluorescence)	PcopA	eGFP*			1.0 μg/L		
	Hg^2+^	mCherry (fluorescence)	Pmer	*mCherry*			0.05 μg/L		
	Cd^2+;^	GFP* (fluorescence)	PzntR	GFP*	*E. coli*	CsgA* amyloid fibrils	0.1–10 μM	not explicitly quantified	[33]
	Cd^2+^	GFP* (fluorescence)	PzntA	GFP*,	*E. coli*	polyacrylamide-alginate hydrogel	0.01 μM	>5 days	[34]
	heme	luminescence	PL(hrto)	*luxCDABE*			~10 μM		
	L-lactate	CreiLOV (fluorescent protein)	PlldR	*CreiLOV*	*E. coli*	hydrogel	5–100 mM	>7 days	[35]
	heme	luminescence	PL(hrto)	*luxCDABE*	*E. coli*	magnetic living hydrogels	~10 μM	>7 days	[36]
	formaldehyde	IAct* (odor)	Pm4	*ATF1*	*E. coli*	porous ceramics	~0.12 ppm	>2 months	[37]
**Light**	light	NanoLuc (luminescence)	PLexA	*NanoLuc*	*S. cerevisiae*	BC	470 nm	>7 days	[38]
	light	YCQ (pro-angiogenic fusion protein)	PFixK2	*YCQ*	*E. coli*	hydrogel	~0.5 μmol·m^−2^·s^−1^	>9 days	[39]
	light	RFP*(fluorescence)	PFixK2	RFP*	*E. coli*	agarose hydrogel	~5 μmol·m^−2^·s^−1^	>4 days	[40]
	light	CsgA*-Mfp3s (adhesive protein)	PFixK2	CsgA*-Mfp3s	*E. coli*	Curli amyloid fibrils	~50 μmol·m^−2^·s^−1^	not explicitly quantified	[41]
	light	deoxyviolacein (anticancer)	PFixK2	*vioABCE*	*E. coli*	hydrogel	~1 μmol·m^−2^·s^−1^	>14 days	[42]
**Other** **Stimulus**	heat	mCherry (fluorescence)	PtlpA39	*mCherry*	*E. coli*	GNC hydrogel	>39 °C	not explicitly quantified	[43]
	heat	β-galactosidase (enzyme for pigment synthesis)	PtlpA36	*LacZ*	*E. coli*	polycarbonate membranes	~42 °C (Repression threshold)	not explicitly quantified	[44]
	mechanical loading	IL-1Ra* (anti-inflammatory protein)	PTGS2r	IL1Ra*	chondrocytes	agarose hydrogels	15% compressive strain	≥3 days	[45]
	electricity	hBMP-4* (osteogenic protein)	PTRE	hBMP-4*	rabbit osteoblasts	PLGA/HA*/PLA-AP/phBMP-4* scaffold	200 mV/cm	≥14 days	[46]

*List of acronyms: aTc, anhydrotetracycline; Ara, arabinose; BC, bacterial cellulose; CsgA, Curli subunit A; GFP, Green Fluorescent Protein; hBMP-4, human Bone Morphogenetic Protein 4; IAct, isoamyl acetate; IL-1Ra, Interleukin-1 Receptor Antagonist; IPTG, Isopropyl β-D-1-thiogalactopyranoside; mCherry, monomeric Cherry fluorescent protein; mtagBFP, monomeric Tag Blue Fluorescent Protein; PGA or γ-PGA, Poly-γ-glutamic acid; PLGA/HA, Poly (lactic-co-glycolic acid)/Hydroxyapatite; RFP, Red Fluorescent Protein; VAI, Vanillic Acid Inducer; YFP, Yellow Fluorescent Protein).

## Data Availability

No new data were created or analyzed in this study. Data sharing is not applicable to this article.

## Abbreviations

The following abbreviations are used in this manuscript:

aTc	anhydrotetracycline;
Ara	arabinose;
AHL	acyl-homoserine lactone;
AuNRs	gold nanorods;
BBC	biofilm@biochar;
BC	bacterial cellulose;
CAP	capsular polysaccharide;
CsgA-Mfp3s	curli fiber fusion protein of CsgA and mussel foot protein 3s;
DOMINO	DNA-based ordered memory and iteration network operator;
dVio	deoxyviolacein;
ELMs	engineered living materials;
EPS	extracellular polymeric substances;
ES	electrical stimulation;
GFP	green fluorescent protein;
hBMP-4	human bone morphogenetic protein 4;
HGT	horizontal gene transfer;
IAct	isoamyl acetate;
IL-1Ra	interleukin-1 receptor antagonist;
IPTG	isopropyl β-D-1-thiogalactopyranoside;
NIR	near-infrared;
PAI	p-coumaric acid inducer;
PGA	poly-γ-glutamic acid;
PLA-AP	poly(l-lactic acid)-block-aniline pentamer-block-poly(l-lactic acid);
PLGA/HA	poly(lactic-co-glycolic acid)/hydroxyapatite;
Plu/PluDA	Pluronic/Pluronic diacrylate
PVD	peripheral vascular disease;
QK	VEGF-mimetic peptide QK;
RFP	red fluorescent protein;
RNAi	RNA interference;
SCRIBE	synthetic cellular recorders integrating biological events;
Tc	tetracycline;
VAI	vanillic acid inducer;
VEGF	vascular endothelial growth factor;
YCQ	YebF-collagen-binding domain-QK fusion protein;
YFP	yellow fluorescent protein.
